# LncRNA LINC00857 strengthens the malignancy behaviors of pancreatic adenocarcinoma cells by serving as a competing endogenous RNA for miR-340-5p to upregulate TGFA expression

**DOI:** 10.1371/journal.pone.0247817

**Published:** 2021-03-04

**Authors:** Tingfu Li, Hongbo Zhao, Hua Zhou, Tingting Geng

**Affiliations:** 1 Central Laboratory, Affiliated Traditional Chinese Hospital of Southwest Medical University, Luzhou, Sichuan, P. R. China; 2 Digest Department, Cent Hospital of Shanxian, Heze, Shandong, P. R. China; 3 Department of Gastroenterology, West Hospital District of Zibo Central Hospital, Zibo, Shandong, P. R. China; 4 Department of Gastroenterology, The Affiliated Yantai Yuhuangding Hospital of Qingdao University, Yantai, Shandong, P. R. China; University of Ulsan College of Medicine, REPUBLIC OF KOREA

## Abstract

**Background:**

Pancreatic adenocarcinoma (PAAD) is a pancreatic disease with a high mortality rate in the world. This present research intends to identify the function of lncRNA LINC00857/miR-340-5p/Transforming growth factor alpha (TGFA) in the progression of PAAD.

**Methods:**

Bioinformatics analysis was used to explore the differentially expressed lncRNA/miRNA/mRNA and analyze the relationship between lncRNA/miRNA/mRNA expression and prognosis of PAAD by enquiring TCGA, GEO and GTE_X_. KEGG pathway analysis and GO enrichment analysis were implemented to annotate the crucial genes regulated by LINC00857. The biological behaviors of PAAD cells were detected by CCK-8, colony formation and transwell assays. Interactive associations between LINC00857 and miR-340-5p, as well as miR-340-5p and TGFA were analyzed by dual luciferase assay.

**Results:**

By enquiring TCGA database, we got that LINC00857 was highly expressed in patients with PAAD and positively associated with worse prognosis in PAAD patients. Moreover, LINC00857 upregulation promoted the proliferation and clone formation abilities of PAAD cells. Afterwards, the downstream miRNA and mRNA targets of LINC00857 were picked up to construct a ceRNA network. Further study revealed that TGFA expression was positively regulated by LINC00857 and negatively regulated by miR-340-5p. Besides that, the inhibitory effect of miR-340-5p on PAAD cells growth and movement can be blocked by LINC00857 upregulation. While, the malignant behavior of PAAD cells induced by TGFA overexpression can be eliminated by LINC00857 knockdown.

**Conclusions:**

Upregulation of LINC00857 improved growth, invasion and migration abilities of PAAD cells by modulation of miR-340-5p/TGFA, affording potential targets and biomarkers for the clinical diagnosis and treatment.

## Introduction

Pancreatic adenocarcinoma (PAAD), as a fatal tumor, is featured by early hematopoietic and lymphocytic hyperplasia following high local recurrence rate [1]. Until now, surgical resection is still the most effective treatment for PAAD. According to statistics, only 18.45% of PAAD patients were diagnosed with stage I or II [2]. Due to the lack of early symptoms and specific molecular markers for early detection and warning, most patients are diagnosed with advanced cancer with local invasion or distant metastasis [3]. The above reasons lead to ineffective surgical resection and drug treatment in PAAD patients, resulting in high mortality and poor prognosis in PAAD patients. In recent years, despite advances in tumor treatment, especially in targeted therapy and immunotherapy, the mortality rate of PAAD is still basically unchanged [4]. Hence, it is essential to further explore the regulatory mechanism during the initiation and progression of PAAD, which may afford an effective solution for the therapy of PAAD.

Long noncoding RNAs (lncRNAs), as non-coding RNAs with a length of over 200 nucleotides, have been discovered to be referred to the development of many diseases [5]. A previous research reported that lncRNAs could act as molecular sponges to compete microRNAs (miRNAs) [6]. For example, lncRNA PVT1 sponged miR-519d-3p to promote progression and glycolysis in pancreatic ductal adenocarcinoma (PDAC) [7]. LncRNA LINC00657 facilitated the malignancy of PDAC via regarding as a competing endogenous RNA (ceRNA) on miR-433 to upregulate PAK4 expression [8]. Additionally, lncRNA ITGB2-AS2 has been discovered to accelerate cell proliferation and aggressiveness via regulating miR-4319/RAF1 in PDAC [9]. In this research, we discovered that LINC00857 was highly expressed in PAAD samples by analyzing the TCGA database. Accordingly, several researches have revealed that LINC00857 played an essential role in numerous tumors, such as lung adenocarcinoma [10], esophageal adenocarcinoma [11], and hepatocellular carcinoma [12]. However, the understanding on the effect of LINC00857 aberrant expression on PAAD is incomprehensive.

It is well known that lncRNAs are able to serve as ceRNAs to regulate the expression of genes by sponging miRNAs [13]. MiRNAs, which composed of 17–24 nucleotides, can control gene expression by targeting to the 3’UTR of the target mRNAs [14]. To date, more than 1500 mature miRNAs were confirmed in the human genome, which can regulate about 50% of all people protein-coding genes [15]. Several literatures revealed that miRNAs are involved in the development of human diseases, including malignancies [16–18]. Currently, abundant miRNAs, such as miR-1290 [19], miR-216b [20], and miR-454 [21], were illustrated to be abnormally expressed in PDAC patients. Significantly, miR-340-5p was served as a downstream target of LINC00857 by visiting bioinformatics software. While, the exact function of miR-340-5p in PAAD cells remains unclear.

Herein, we used biological information analysis to explore the expression of LINC00857 in PAAD and the prognostic relationship with PAAD patients. Besides, we performed functional experiments to detect the biological properties of LINC00857 in the malignant progression of PAAD cells and the relevant downstream molecular mechanisms.

## Materials and methods

### Data acquisition and bioinformatics analysis

The association between LINC00857 and miR-340-5p was speculated by lncbase2.0 website. Targetscan database was used to explore the upstream regulator of Transforming growth factor alpha (TGFA). TCGA (https://cancergenome.nih.gov/, project-PAAD) and GTEx database were applied to analyze the expression of LINC00857 and TGFA in PAAD patients. TCGA and Gene expression omnibus (GEO) database were applied to detect miR-340-5p expression in PAAD patients. Kyoto Encyclopedia of Genes and Genomes (KEGG) pathway analysis and Gene Ontology (GO) enrichment analysis were implemented to annotate the crucial genes, which were co-regulated with LINC00857. OncomiR website was used to select the differentially expressed miRNA in PAAD samples.

### Cell source and culture

Two human PAAD cell lines (PANC-1 or SW 1990) were awarded from the Cell Bank of the Chinese Academy of Sciences (Shanghai, China). The normal pancreatic cell line (HPDE6-C7) was purchased from American Type Culture Collection (ATCC, Manassas, USA). The cells were cultivated in Dulbecco’s modified Eagle’s medium (DMEM) covering 10% fetal bovine serum (FBS), 100 U/mL penicillin, and 100 mg/mL streptomycin (Sigma, USA) under the condition of 37°C, 5% CO_2_.

### Regulation of genes expression

Firstly, PANC-1 or SW 1990 cells were implanted into six-well plates with a fresh density of 1×10^5^ cells per well. When the cells covered 60% confluence, pcDNA3.1-LINC00857/si-LINC00857, miR-340-5p mimic/inhibitor, pcDNA3.1-TGFA/si-TGFA were transfected into cells to respectively elevate or reduce LINC00857, miR-340-5p and TGFA expression. All the transfection experiments were performed with the support of Lipofectamine 2000 (Invitrogen, USA).

### qPCR

TRIzol reagent (Beyotime, China) was used to isolate the whole RNA from the treated PANC-1 or SW 1990 cells. To analyze miR-340-5p expression, the cDNA was generated from the whole RNA with the assistance of miScript reverse transcription kit, and qPCR was carried out with the help of miScript SYBR Green PCR kit. U6 functioned as the internal standard for miR-340-5p detection. For the evaluation of LINC00857 and TGFA expression, the whole RNA was converted into cDNA using PrimeScript^™^ RT Reagent Kit. Afterwards, qPCR was implemented by utilizing the SYBR-Green PCR Master Mix. All the procedures were performed on ABI 7500 real time PCR system. GAPDH was served as the internal standard for LINC00857 and TGFA detection. 2^−ΔΔCt^ method was employed to evaluate the relative genes expression. The primers were constructed as follows:

LINC00857 Forward: 5’-TGAATAGCTACCCCTGGGCT-3’, Reverse: 5’-AGCCCAGGGGTAGCTATTCA-3’; miR-340 Forward: 5’- TATAAAGCAATGAGACTGAT-3’, Reverse: 5’-GAACATGTCTGCGTATCTC-3’; U6 Forward: 5’-CTCGCTTCGGCAGCACAT-3’, Reverse: 5’-TTTGCGTGTCATCCTTGCG-3’; TGFA Forward: 5’-GGTCCGAAAACACTGTGAGTGG-3’, Reverse: 5’- CAAACTCCTCCTCTGGGCTCTT-3’; GAPDH Forward: 5’- CCATGGGGAAGGTGAAGGTC-3’, Reverse: 5’-AAATGAGCCCCAGCCTTCTC-3’.

### Detection of protein expression

Western blot was applied to examine the related protein expression levels. Firstly, transfected PANC-1 or SW 1990 cells were washed by pre-cooling PBS and then lysed using RIPA buffer (1% protease inhibitor) to extract protein. The concentration of protein was determined by a BCA protein assay kit. Afterwards, the protein was separated through SDS-PAGE gel and blotted onto PVDF membranes. After sealing with 5% skimmed milk, the membranes were incubated with primary antibodies against TGFA or GAPDH, which were obtained from Abcam (Cambridge, MA, USA). Subsequently, the membranes were incubated with the secondary antibodies. Protein bands were developed after suffered from ECL and quantified with Image J software.

### Determination of cell activity

The proliferation of PANC-1 or SW 1990 cells was examined with the assistance of CCK-8 assay. After 24 h post-transfection, the cells were collected. Afterwards, the processed single cell suspension was seeded in 96-well plates with a density of 2000 cells per well. The CCK-8 assay was implemented at 0, 24, 48 and 72 h. After cell seeding, 15 μL of CCK-8 reagent was loaded into per well, accompanied by incubation at same condition for another 1.5 h. The optical density (OD) value was examined at 450 nm on a microplate reader.

### Cell invasion and migration experiments

Invasive and migratory abilities of treated PANC-1 or SW 1990 cells were explored adopting transwell chambers. Firstly, treated cells were rinsed with PBS and re-suspended in DMEM excluding FBS. 200 μL cell suspension including 1×10^5^ cells was added into the above chamber. The lower chamber was loaded with 500 μL of DMEM including 10% FBS. After 24 h of incubation, get rid of the non-migratory cells by using a cotton swab, while the migratory cells were fixed with paraformaldehyde and stained with crystal violet. The migratory cells images were caught using a microscope. The procedures of the invasion experiment were the same to the migration assay, except that the chambers were pre-coated with Matrigel.

### Plate clone formation assay

For this experiments, 500 PANC-1 or SW 1990 cells were seeded onto the six-well plates with DMEM (10% FBS), each group consisting of 3 holes. After 10–14 days cultivation, the clones were rinsed twice with PBS and stained with crystal violet staining (Beyotime, China). Finally, a microscope was applied to count the colony forming units.

### Luciferase report assay

The wild-type (wt) LINC00857/TGFA with the predicting binding sites of miR-340-5p was cloned and inserted into the pGL3-vector, named as wt- LINC00857/TGFA. While, the mutant (mut) LINC00857/TGFA sequence was synthesized and inserted into pGL3-vector, called mut-LINC00857/TGFA. Afterwards, the wt-LINC00857/TGFA or mut-LINC00857/TGFA and miR-340-5p mimic/inhibitor were together transfected into cells by using Lipofectamine 2000 (Invitrogen, USA). After cultivation for 48 h, the cells were gathered and the luciferase activity was examined by using the dual luciferase reporter assay system based on the supplier’s instruction.

### Data processing

The relationship between LINC00857 and clinical properties of PAAD patients was analyzed by using the chi-square test. Cox regression analysis was used to explore the independent prognostic factor of PAAD patients. Pearson’s correlation analysis was executed to evaluate the expression relationship between LINC00857 and TGFA. Student’s t test was applied to analyze the comparison between two groups, whereas ANOVA along with Bonferroni’s post hoc test was implemented to estimate the difference among multiple groups. Experimental values, which were from three independent experiments, were exhibited as mean ± standard deviation through SPSS22.0 and Graphpad Prism 5.0 analysis. P < 0.05 was regarded as statistically significant.

## Results

### LINC00857 is upregulated in PAAD samples and contributes to malignant behavior in PAAD cells

**Fig 1:**
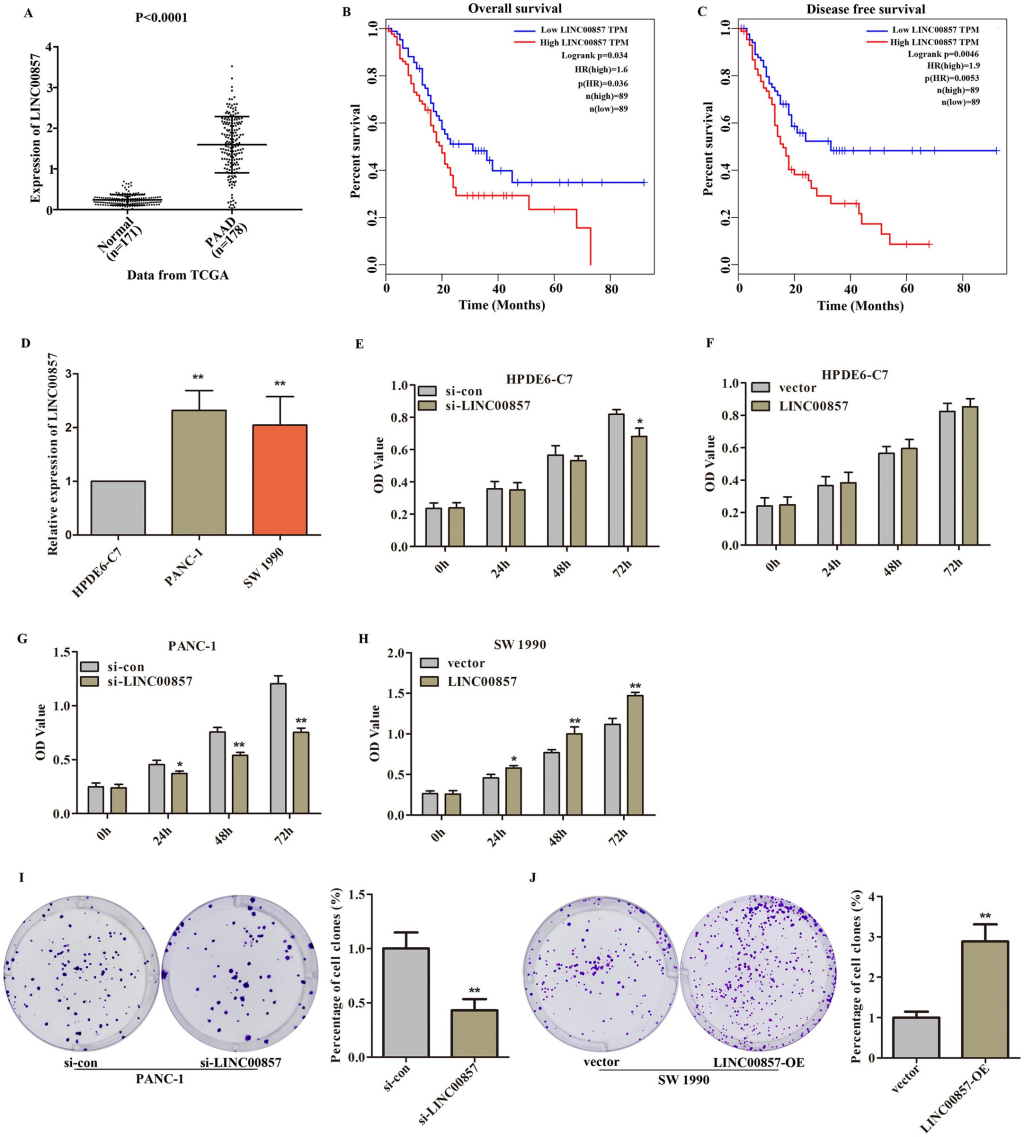
The biological function of LINC00857 in PAAD was discovered. A. The expression level of LINC00857 was identified by enquiring TCGA and GTEx database, including 178 PADD patients and 171 normal samples, P<0.001. B. Overexpression of LINC00857 significantly reduced the overall survival of PAAD, P = 0.034. C. The disease free survival of PAAD patients significantly decreased with high expression of LINC00857, P = 0.0046. D. LINC00857 was obviously upregulated in PANC-1 and SW 1990 cells compared with HPDE6-C7 cells, **P<0.01 vs. HPDE6-C7. E. The influence of si-LINC00857 on HPDE6-C7 cells proliferation was analyzed by CCK-8 assay, *P<0.05 vs. si-con. F. Upregulation of LINC00857 has no effect on HPDE6-C7 cells proliferation. G. Knockdown of LINC00857 reduced PANC-1 cells viability, *P<0.05, **P<0.01 vs. si-con. H. LINC00857 overexpression caused an increase in SW 1990 activity, *P<0.05, **P<0.01 vs. vector. I. LINC00857 depletion induced a decrease in PANC-1 cells colony formation ability, **P<0.01 vs. si-con. J. LINC00857 upregulation promoted SW 1990 cells colony formation ability, **P<0.01 vs. vector.

Firstly, data from TCGA and GTEx, which including 178 PADD patients and 171 normal samples, showed that LINC00857 was highly expressed in patients with PAAD. Moreover, its high expression led to a shorter survival time for PADD patients (Fig 1A and 1B, P<0.05). With the increase of LINC00857 expression, the total survival time of PAAD patients decreased significantly (Fig 1C, P = 0.0053), which further confirmed the importance of LINC00857 in the prognosis of PDAA patients. Afterwards, we explored the clinical value of LINC00857 in PAAD by estimating the relationship between LINC00857 expression and clinical properties of the 162 cases of PADD patients with complete clinical information. The results revealed that the expression of LINC00857 was associated with Death in PAAD patients, which was statistically significant (Table 1). After Cox regression analysis of the samples, it was found that LINC00857 could be used as an independent predictor of the prognosis of PAAD (Table 2).

**Table 1:** Relationship between LINC00857 expression and clinicopathological properties of patients with pancreatic adenocarcinoma.

Characteristics	Expression of LINC00857		P value
	Low	High	
**Age**			0.231
<60	21	28	
≥60	60	53	
**Gender**			0.344
female	34	40	
male	47	41	
**Grade**			0.392
G1+G2	59	54	
G3+G4	22	27	
**Stage**			0.699
I+II	78	77	
III+IV	3	4	
**T-Stage**			0.277
T1+T2	15	10	
T3+T4	66	71	
**N**			0.861
N0	22	23	
N1	59	58	
**M**			1.000
M0	79	79	
M1	2	2	
**Death**			0.002*
No	47	27	
Yes	34	54	

*P<0.05, T: tumor, N: lymph nodes, M: metastasis.

**Table 2:** Cox univariate and multivariate analysis of LINC00857 in pancreatic adenocarcinoma patients.

Variables	Univariate analysis			Multivariate analysis		
	Pvalue	HR	95%CI	Pvalue	HR	95%CI
**LINC00857 expression**(high/low)	0.013*	1.720	1.119–2.643	0.020*	1.665	1.083–2.561
**Stage** (I+II/III+IV)	0.724	0.812	0.256–2.578			
**T-stage** (T1+T2/T3+T4)	0.076	1.819	0.938–3.528			
**M** (M0/M1)	0.992	1.007	0.247–4.114			
**N** (N0/N1)	0.005*	2.158	1.269–3.670	0.006*	2.096	1.231–3.568
**Age**(<60/≥60)	0.244	1.329	0.824–2.144			
**Gender** (female/male)	0.208	0.763	0.501–1.163			
**Grade**(G1+G2/G3+G4)	0.097	1.451	0.935–2.249			

*P<0.05, T: tumor, N: lymph nodes, M: metastasis.

Subsequently, we detected LINC00857 expression in PAAD cell lines including PANC-1 and SW 1990. The result from PCR showed that LINC00857 expression was obviously elevated in PANC-1 and SW 1990 cells compared with that of HPDE6-C7 cells (Fig 1D, P<0.01). Furthermore, the CCK-8 assay showed that depletion of LINC00857 obviously reduced the OD values of PANC-1 cells (Fig 1G, P<0.05). However, the OD values of HPDE6-C7 cells was not affected by LINC00857 depletion until 72 h (Fig 1E, P<0.05). Moreover, the plate clone assay revealed that knockdown of LINC00857 reduced the cloning formation number of PANC-1 cells (Fig 1I, P<0.01). After upregulation of LINC00857, the OD values of SW 1990 cells were elevated obviously (Fig 1H, P<0.05). However, the OD values of HPDE6-C7 cells was not affected by LINC00857 upregulation (Fig 1F). Additionally, after pcDNA3.1-LINC00857 stimulation, the cloning formation number of SW 1990 cells was increased significantly (Fig 1J, P<0.05). These observations revealed that LINC00857 functioned as a key regulator in PAAD progression.

### LINC00857 is positively related with TGFA, a target of miR-340-5p

**Fig 2:**
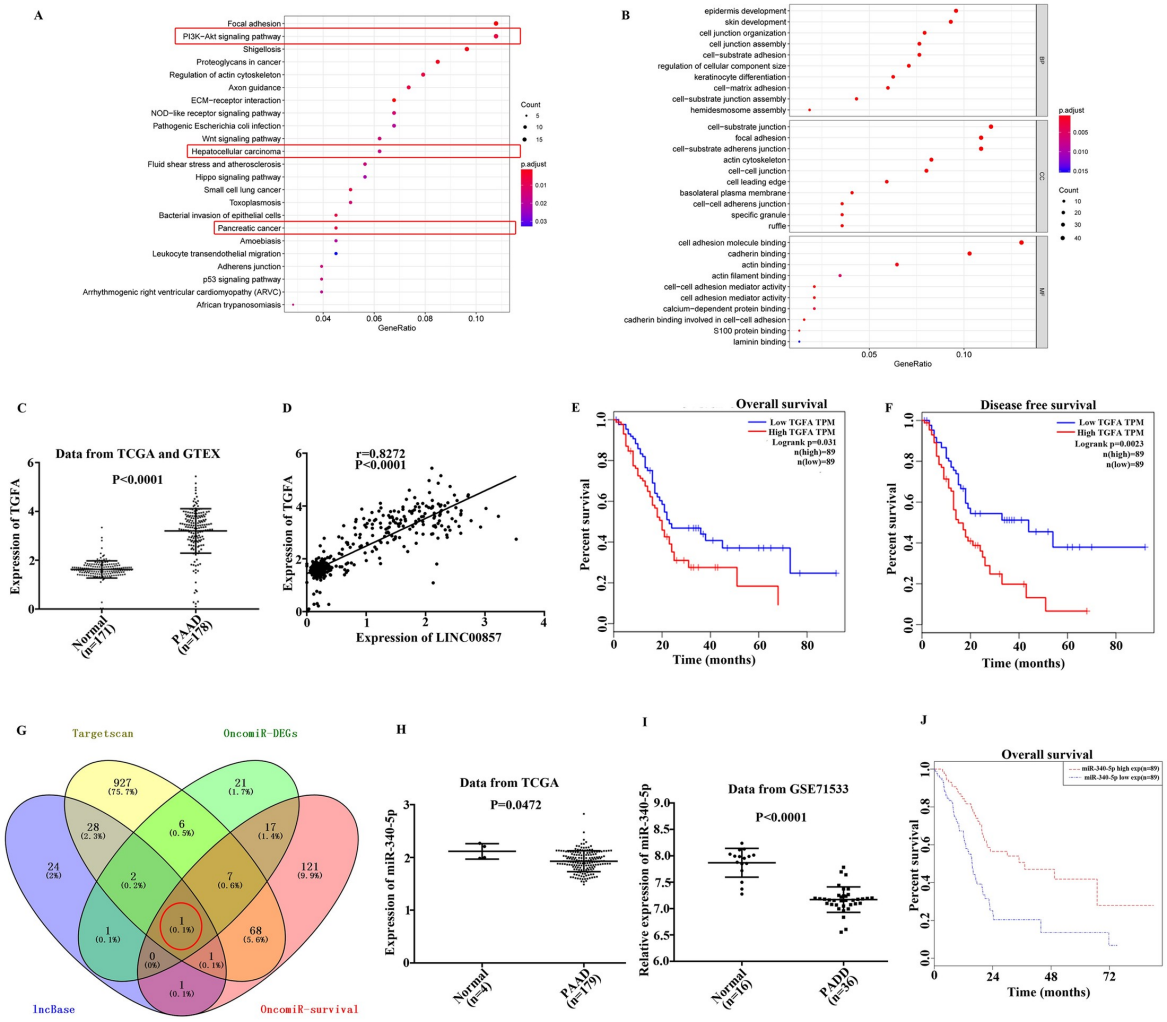
The potential ceRNA mechanism was constructed. A, B. KEGG pathway analysis and GO enrichment analysis were applied to detect the signaling pathway related to the 257 differentially expressed genes, TGFA was related to PI3K-AKT pathway, Hepatocellular carcinoma, and Pancreatic cancer. C. TGFA expression was significantly increased in PAAD patients, P<0.0001. D. A positive association was existed between LINC00857 and TGFA expression, P<0.0001. High expression of TGFA led to poor overall survival (E, P = 0.031) and disease free survival (F, P = 0.0023). G. miR-340-5p was picked up by intersection of four datasets. H. Lower expression of miR-340-5p was occurred in 179 PAAD patients from TCGA database, P = 0.0472. I. Data from GEO including 36 cases of PAAD patients and 16 normal samples revealed that miR-340-5p was lower expressed in PAAD, P<0.0001. J. Lower expression of miR-340-5p contributed to bad overall survival of PAAD patients, P<0.05.

Based on the above findings, mRNAs with strong co-expression with LINC00857 were selected by KEGG and GO enrichment analysis. It was found that TGFA was widely involved in the signaling pathways (PI3K-AKT pathway, Hepatocellualr carcinoma, Pancreatic cancer) from KEGG and was significantly upregulated in PAAD patients (n = 178) than the normal samples (n = 171, Fig 2A–2C, P<0.05). And a positive relationship between LINC00857 and TGFA expression was identified by Pearson’s correlation analysis (Fig 2D, P<0.0001). Moreover, with the increase of TGFA expression, the overall survival and disease-free survival of patients with high expression were lower than those with TGFA low expression, indicating that TGFA was an adverse prognostic factor in PADD patients (Fig 2E and 2F, P<0.05).

**Fig 3:**
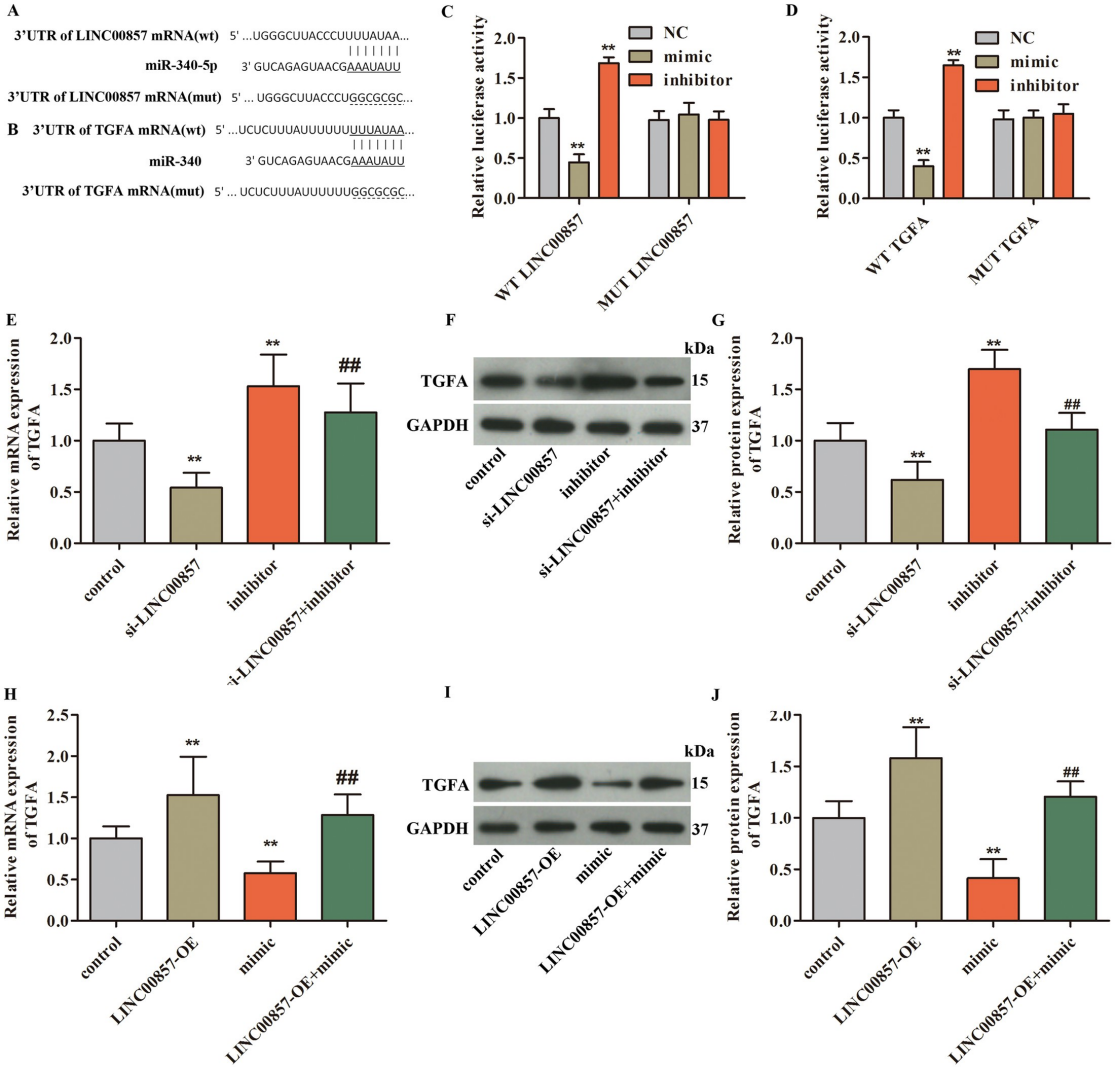
Associations among LINC00857, miR-340-5p, and TGFA were identified. A. The binding sites between LINC00857 and miR-340-5p were presented. B. The binding sequences between miR-340-5p and TGFA were exhibited. C. The luciferase activity in WT-LINC00857 group was reduced by miR-340-5p mimic treatment, but miR-340-5p inhibitor increased the luciferase activity in WT-LINC00857 group, **P<0.01 vs. control. D. miR-340-5p mimic decreased the luciferase activity in WT-TGFA group, while miR-340-5p inhibitor increased the luciferase activity in WT-TGFA group, **P<0.01 vs. control. E-G. The mRNA and protein levels of TGFA in PCNA-1 cells were reduced after si-LINC00857 treatment, miR-340-5p treatment increased TGFA expression, **P<0.01 vs. control, ^##^P<0.01 vs. si-LINC00857, ^&&^P<0.01 vs. miR-340-5p inhibitor. H-J. In SW 1990 cells, TGFA mRNA and protein expression was increased when LINC00857 upregulation, but overexpression of miR-340-5p suppressed TGFA expression, **P<0.01 vs. control, ^##^P<0.01 vs. LINC00857-OE, ^&&^P<0.01 vs. miR-340-5p mimic.

Thereafter, the miRNA related to LINC00857 and TGFA was explored. The target miRNA of LINC00857 (lnc Base, purple), the upstream miRNA regulator of TGFA (Targetscan, yellow), the down-regulated miRNA in PAAD obtained from the OncomiR website (OncomiR-DEGs, green), and the miRNAs that were meaningful for survival (OncomiR-survival) were intersected to obtain a common miRNA (orange). Finally, miR-340-5p is screened out (Fig 2G). The data from TCGA presented that lower miR-340-5p expression, which led to bad survival of PAAD patients, was observed in PAAD patients (Fig 2G and 2H, P<0.05). The binding sequences between LINC00857 and miR-340-5p, as well as miR-340-5p and TGFA were presented in Fig 3A and 3B. Moreover, the data from dual luciferase assay indicated that the luciferase activity in WT-LINC00857 group was reduced or increased after miR-340-5p mimic or inhibitor treatment. Similarly, in WT-TGFA group, the luciferase activity was declined or elevated with miR-340-5p mimic or inhibitor stimulation. However, in MUT-LINC00857 or MUT-TGFA group, the luciferase activity has no change (Fig 3C and 3D, P<0.01). Furthermore, the data from qPCR and western blot suggested that TGFA expression was positively correlated with LINC00857 and negatively related to miR-340-5p. These discoveries insinuated that LINC00857, miR-340-5p and TGFA may construct a ceRNA network to regulate PAAD cells behaviors.

### LINC00857, miR-340-5p, and TGFA co-regulate PAAD cells growth and aggressiveness

**Fig 4:**
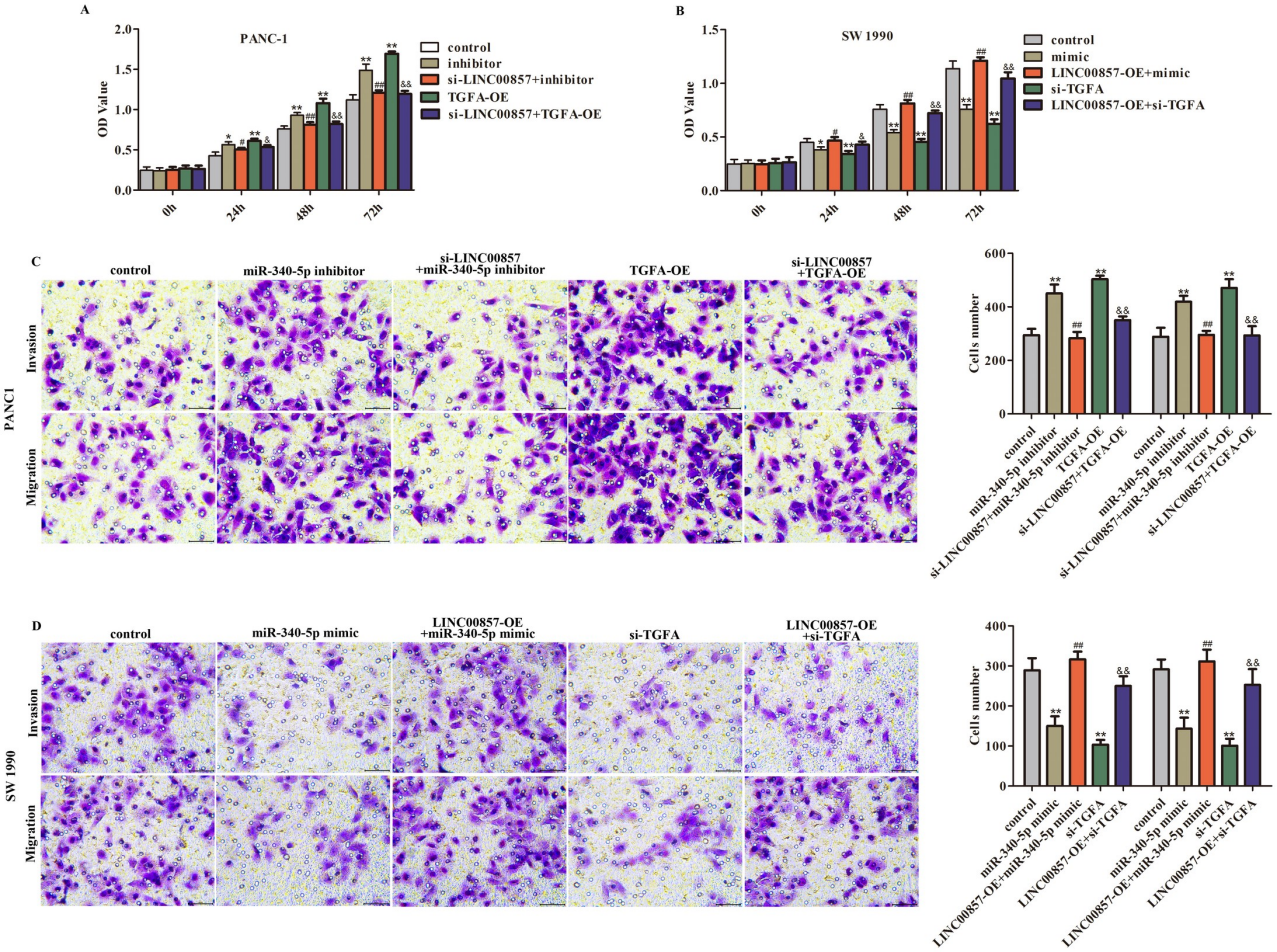
Function of miR-340-5p and TGFA on PAAD cells was regulated by LINC00857. A. The increased OD values of PANC-1 cells caused by miR-340-5p inhibitor or TGFA-OE was suppressed by LINC00857 depletion. B. LINC00857 upregulation inversed the decreased OD values of SW 1990 cells induced by miR-340-5p mimic or si-TGFA on SW 1990 cells proliferation. C. miR-340-5p inhibitor or TGFA-OE treatment increased the invaded and migrated number of PANC-1 cells, but depletion of LINC00857 reversed these phenomena. D. miR-340-5p mimic or si-TGFA treatment reduced the invaded and migrated number of SW 1990 cells, but LINC00857 upregulation eliminated this trend. **P<0.01 vs. control, ^##^P<0.01 vs. miR-340-5p inhibitor/mimic, ^&&^P<0.01 vs. TGFA-OE/si-TGFA.

Next, CCK-8 and transwell assays were implemented to explore the function of LINC00857, miR-340-5p, and TGFA on the proliferation and aggressiveness of PANC-1 and SW 1990 cells. The data suggested that depletion of miR-340-5p or overexpression of TGFA elevated the PANC-1 cells growth and aggressiveness abilities. However, these positive effects on PANC-1 cells caused by miR-340 inhibitor or TGFA-OE can be eliminated by LINC00857 depletion (Fig 4A and 4C, P<0.01). Whilst, the growth and aggressiveness abilities of SW 1990 cells were decreased after miR-340-5p overexpression or TGFA depletion. However, the decreasing trends of SW 1990 cells growth and aggressiveness induced by miR-340 mimic or si-TGFA can be alleviated by LINC00857 upregulation (Fig 4B and 4D, P<0.01). These phenomena illustrated that the function of miR-340-5p or TGFA on PAAD cells can be regulated by LINC00857, indicating that LINC00857, miR-340-5p, and TGFA formed a ceRNA network to regulate the biological behaviors of PAAD cells.

## Discussion

Currently, the expression levels and function of lncRNAs in PAAD were detected widely [22, 23]. Increasing lncRNAs have been observed to be aberrantly expressed in PAAD, which played crucial roles in the initiation and progression of PAAD [24, 25]. So, illuminating function of cancer-related lncRNAs in PAAD pathogenesis may promote the identification of promising targets for the treatment of PAAD patients. This research revealed that LINC00857 was highly expressed in PAAD patients and positively correlated with short-term survival of PAAD patients. Additionally, the function of LINC00857 on the malignant behaviors of PAAD cells and potential molecular mechanism was uncovered.

Anteriorly, LINC00857 was considered as one of the most upregulated lncRNAs in lung cancer. LINC00857 depletion has been discovered to block cell proliferation and metastasis by modulating a series of genes implicated in cell cycle progression. Significantly, similarly to what we have discovered in PAAD, LINC00857 upregulation was connected with shorter survival time of patients with lung cancer, indicating its oncogenic role in cancer [26]. Besides that, LINC00857 expression in the primary tumor was presented to predict muscle-invasive bladder cancer development and response to chemotherapy [27]. Furthermore, LINC00857 depletion blocked cell growth and promoted apoptosis in esophageal adenocarcinoma [11]. In our study, LINC00857 was highly expressed in PAAD patients and cell lines, and LINC00857 upregulation significantly related to the death with the PAAD patients. Through clinical correlation analysis, we observed that LINC00857 expression has no obvious relationship with the patient’s pathological stage, metastasis and other characteristics. The possible reason is that the sample size of patients from TCGA is small, which has some limitations. Moreover, LINC00857 can be used as an independent prognostic factor for PAAD. More importantly, overexpression of LINC00857 significantly elevated the viability and clone formation abilities of PADD cells.

The lncRNA-mediated biological properties of cancer cells are based on the crosstalk between lncRNAs and mRNAs, which compete for shared response elements in miRNAs [28]. This ceRNA mechanism, which has been extensively discovered in tumors, has attracted widespread interest [29, 30]. For example, LINC00857 modulated lung adenocarcinoma progression by targeting miR-1179/SPAG5 [10]. In this research, the mechanism related to the oncogenic activity of LINC00857 in PAAD cells was explored. By informatics analysis, we discovered that TGFA was widely existed in the KEGG enrichment pathway and has a strong co-expression relation with LINC00857, indicating that TGFA was a potential downstream mRNA of LINC00857. In addition, TGFA was also highly expressed in PAAD patients and was significantly related to poor prognosis of PAAD patients. TGFA, which encodes a growth factor that is a ligand for the epidermal growth factor receptor, was positively related to cell growth, migration, and development [31]. In addition, TGFA has been illustrated to be positively related to several cancers, such as prostate cancer [32], colon cancer [33], and breast cancer [34]. Consistent with the reports in the above literatures, our study observed that TGFA overexpression increased the PAAD cells growth and aggressiveness abilities. However, LINC00857 depletion blocked the promoting effect of TGFA on PAAD cells malignant behaviors, insinuating a potential relationship between LINC00857 and TGFA.

To construct the ceRNA network between LINC00857 and TGFA in PAAD, miR-340-5p was picked up as the most suitable miRNA. As a key molecule linking LINC00857 and TGFA, miR-340-5p has attracted extensive attentive in human cancer. For example, miR-340-5p was lower expressed in colorectal cancer and acted as a tumor suppressor in colorectal cancer by binding ANXA3 [35]. While, in thyroid cancer, miR-340-5p was highly expressed and executed an active role [36]. Consistent with the function in colorectal cancer, we discovered that miR-340-5p was lower expressed in PAAD patients and lower expression of miR-340-5p had a worse overall survival in patients with PAAD. Importantly, upregulation of miR-340-5p limited the malignant behaviors of PAAD cells. However, this effect can be blocked by LINC00857 overexpression, indicating an underlying association between LINC00857 and miR-340-5p.

Our study discovered the pro-carcinogenic effect of LINC00857 in PAAD cancer cells, that is, LINC00857 was implicated in the malignancy of PAAD via acting as a ceRNA competitively targeting to miR-340-5p and thereby upregulating TGFA expression. The findings provided potential molecules for targeted therapy of PAAD.

## Supporting information

S1 File (XLSX)

S1 Raw images (DOC)
