# Supplementary material for: LncRNA LINC00857 strengthens the malignancy behaviors of pancreatic adenocarcinoma cells by serving as a competing endogenous RNA for miR-340-5p to upregulate TGFA expression
Source: PLoS One. 2021 Mar 4;16(3):e0247817. doi: 10.1371/journal.pone.0247817 (PMC7932076; doi:10.1371/journal.pone.0247817)
Supplement: S1 Raw images — (DOC) [file pone.0247817.s002.doc]

Dear editor, we have added the original protein bands as below. Since we cut the membranes after the transfer was completed during the experiment, there was no complete membranes. However, to demonstrate the size of our target protein, we added the following image. Please check.

The image from fig. 3F (TGFA)

15kDa

The image from fig. 3F (GAPDH)

37kDa

The image from fig. 3I (TGFA)

15kDa

The image from fig. 3I (GAPDH)

37kDa
